# Genome skimming yields the complete mitogenome of *Chromodoris annae* (Mollusca: Chromodorididae)

**DOI:** 10.1080/23802359.2017.1372715

**Published:** 2017-09-05

**Authors:** Geng-Ming Lin, Peng Xiang, Bonifasius Putera Sampurna, Chung-Der Hsiao

**Affiliations:** a Laboratory of Marine Biology and Ecology, Third Institute of Oceanography, SOA, Xiamen, China;; b Department of Bioscience Technology, Chung Yuan Christian University, Chung-Li, Taiwan;; c Center for Biomedical Technology, Chung Yuan Christian University, Chung-Li, Taiwan;; d Center for Nanotechnology, Chung Yuan Christian University, Chung-Li, Taiwan

**Keywords:** Sea slug, mitogenome, next generation sequencing

## Abstract

In this study, the complete mitogenome sequence of sea slug, *Chromodoris annae* (Mollusca: Chromodorididae), has been decoded for the first time by genome skimming method. The overall base composition of *C. annae* mitogenome is 30.6% for A, 14.5% for C, 17.8% for G, and 37.2% for T, and has GC content of 32.2%. The assembled mitogenome, consisting of 14,260 bp, has unique 13 protein-coding genes (PCGs), 22 transfer RNAs, and two ribosomal RNAs genes. The *C. annae* has the common mitogenome gene organization and feature of Chromodorididae. The complete mitogenome of *C. annae* provides essential and important DNA molecular data for further phylogenetic and evolutionary analysis for sea slugs.

*Chromodoris annae* (Mollusca: Chromodorididae) is a beautiful sea slug which is characterized morphologically by thin body wall, lack of specular element in the integument, resulting in very soft and deformable body (Willan 2010). *C. annae* feeds on one group of chemically noxious sponge and adsorbs as its defence mechanism, stores in the glands of the mantle. The active chemicals from these sponges are distasteful to fish (Harvell and Greene 2016). This species is widely distributed in tropical region specifically in Indo-West Pacific Ocean from Malaysia to Marshall Islands, with depth range 15–30 m. *C. annae* is one of the quadricolor group of *Chromodoris*. The body has a blue pigment arranged in a reticule pattern with dark specks, the space appearing in translucent grey and orange border. In earlier publication, *C. annae* was confused considering to be synonymous with *C. elisabethina* (Rudman 1982). Morphological approach can lead to a confusing justification to distinguish between the individuals. Mitogenome is one of the molecular aspect that can be used to discriminate the species of one individual. Complete mitogenome can be combined with morphological assay to verify the assessment of the sea slug with more higher resolution.

Samples (voucher no. 642 kept in Chung Yuan Christian University) of *C. annae* were collected from local aquarium in Taiwan and originally imported from Bali island of Indonesia (8°27′S 115°4′E). The methods for genomic DNA extraction, library construction, and next generation sequencing were described in our previous publication (Shen et al. 2016). Initially, the raw next generation sequencing reads generated from HiSeq X Ten (Illumina, San Diego, CA) were filtered to remove low quality reads. Around 0.05% raw reads (8592 out of 16,959,774) were subjected to *de novo* assembly using commercial software (Geneious V9, Auckland, New Zealand) to produce a single, circular form of complete mitogenome with about an average 90× coverage. The complete mitogenome of *C. annae* contains 14,260 bp in size (GenBank MF683074) with overall base composition of 30.6% for A, 14.5% for C, 17.8% for G, and 37.2% for T, and has GC content of 32.2%, showing 99% identities to the complete mitogenome of *C. quadricolor* (GenBank KU317089) which is reported in our previous study (Xiang et al. 2016).

The protein coding, rRNA and tRNA genes of *C. annae* mitogenome were predicted by using DOGMA (Wyman et al. 2004), ARWEN (Laslett and Canback 2008), and MITOS (Bernt et al. 2013) tools, and manually inspected. The complete mitogenome of *C.* includes unique 13 protein-coding genes (PCGs), 22 transfer RNA genes, and two ribosomal RNA genes. The *C. annae* mitogenome has the common gene organization and feature with other Chromodorididae species. The longest gene of all PCGs is ND5 gene (1623 bp), whereas the shortest one is ATP8 gene (165 bp). The size of small ribosomal RNA (12S rRNA) and large ribosomal RNA (16S rRNA) genes is 744 bp and 1098 bp, respectively. The size of all tRNA ranged from 55 to 69 bp. Four PCGs (ATP8, ND3, ATP6, and COX3), one rRNA (12S rRNA), and eight tRNA (tRNA-Ser, tRNA-Gln, tRNA-Arg, tRNA-Leu, tRNA-Glu, tRNA-Met, tRNA-Thr, and tRNA-Asn) genes are encoded on reversed-strand, while other genes are encoded in the forward-strand.

To validate the phylogenetic position of *C. annae,* we used MEGA6 software (Tamura et al. 2013) to construct a Maximum likelihood tree (with 500 bootstrap replicates) containing complete mitogenomes of nine species derived from Nudibranchia. *Aplysia californica* derived from Aplysiidae was used as outgroup for tree rooting. Result shows *C. annae* is close to *C. quadricolor* and unambiguously grouped with other *Chromodoris* species having high bootstrap value supported (Figure 1). In conclusion, the complete mitogenome of the *C. annae* deduced in this study provides essential and important DNA molecular data for further phylogenetic and evolutionary analysis for sea slug.

**Figure 1.**
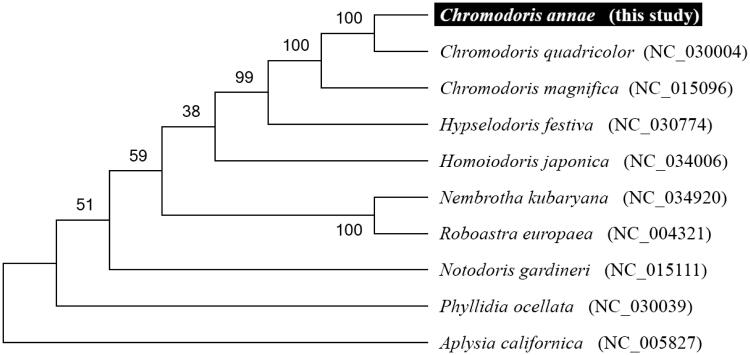
Molecular phylogeny of *Chromodoris annae* and related species in Nudipleura based on complete mitogenome. The complete mitogenomes are downloaded from GenBank and the phylogenic tree is constructed by maximum likelihood method with 500 bootstrap replicates. The gene's accession number for tree construction is listed behind species name.
